# Kinetic and Stoichiometric Modeling-Based Analysis of Docosahexaenoic Acid (DHA) Production Potential by *Crypthecodinium cohnii* from Glycerol, Glucose and Ethanol

**DOI:** 10.3390/md20020115

**Published:** 2022-02-01

**Authors:** Kristaps Berzins, Reinis Muiznieks, Matiss R. Baumanis, Inese Strazdina, Karlis Shvirksts, Santa Prikule, Vytautas Galvanauskas, Daniel Pleissner, Agris Pentjuss, Mara Grube, Uldis Kalnenieks, Egils Stalidzans

**Affiliations:** 1 Institute of Microbiology and Biotechnology, University of Latvia, Jelgavas Street 1, LV-1004 Riga, Latvia; kristaps.berzins@lu.lv (K.B.); reinis.muiznieks@lu.lv (R.M.); matiss.baumanis@gmail.com (M.R.B.); inese.strazdina@lu.lv (I.S.); karlis.svirksts@lu.lv (K.S.); santaprikule@gmail.com (S.P.); agris.pentjuss@gmail.com (A.P.); mara.grube@lu.lv (M.G.); uldis.kalnenieks@lu.lv (U.K.); 2 Biotehniskais Centrs AS, Dzerbenes Street 27, LV-1006 Riga, Latvia; vytautas.galvanauskas@ktu.lt; 3 Department of Automation, Kaunas University of Technology, LT-51367 Kaunas, Lithuania; 4 Sustainable Chemistry (Resource Efciency), Institute of Sustainable and Environmental Chemistry, Leuphana University of Lüneburg, Universitätsallee 1, C13.203, 21335 Luneburg, Germany; daniel.pleissner@leuphana.de; 5 Institute for Food and Environmental Research (ILU), Papendorfer Weg 3, 14806 Bad Belzig, Germany

**Keywords:** Krebs cycle, central metabolism, kinetic model, constraint-based model, FTIR spectroscopy

## Abstract

Docosahexaenoic acid (DHA) is one of the most important long-chain polyunsaturated fatty acids (LC-PUFAs), with numerous health benefits. *Crypthecodinium cohnii*, a marine heterotrophic dinoflagellate, is successfully used for the industrial production of DHA because it can accumulate DHA at high concentrations within the cells. Glycerol is an interesting renewable substrate for DHA production since it is a by-product of biodiesel production and other industries, and is globally generated in large quantities. The DHA production potential from glycerol, ethanol and glucose is compared by combining fermentation experiments with the pathway-scale kinetic modeling and constraint-based stoichiometric modeling of *C. cohnii* metabolism. Glycerol has the slowest biomass growth rate among the tested substrates. This is partially compensated by the highest PUFAs fraction, where DHA is dominant. Mathematical modeling reveals that glycerol has the best experimentally observed carbon transformation rate into biomass, reaching the closest values to the theoretical upper limit. In addition to our observations, the published experimental evidence indicates that crude glycerol is readily consumed by *C. cohnii,* making glycerol an attractive substrate for DHA production.

## 1. Introduction

Knowledge-based bioeconomy implies the conversion of cheap renewable resources into biotechnological products with added value.

Docosahexaenoic acid (DHA) is one of the most important long-chain polyunsaturated fatty acids (LC-PUFAs), with numerous health benefits such as reducing the risk of cardiovascular diseases, cancer, and rheumatoid arthritis; alleviating depression symptoms and post-natal depression; and contributing to immune-modulatory effects [1]. DHA also has an important role in the healthy development of the fetal brain and retina, and thus is commonly used in infant-related food products. The global EPA/DHA market was estimated at USD 2.49 billion in 2019, with a projected annual growth rate of 7% until 2027 [2]. Currently, cold-water marine fish oil is a source of 96% of DHA, but it is not able to meet the increasing demand of DHA for human consumption [3] due to the depletion of wild fish stocks and pollution of the marine environment (with lipophilic environmental pollutants, dioxins, heavy metals, etc.). Moreover, fish and other animals lack certain fatty acid desaturases that are required for the de novo synthesis of LC-PUFAs. Plants, although a commercially important source of oils and fats, do not synthesize LC-PUFAs.

Efforts to explore alternative sources of DHA have been made in the last decade, including the generation of transgenic oilseed plants [4] and large-scale production of DHA-producing microalgae and protists [5]. As microbes synthesize all of their cell lipid fatty acids de novo, the profile of these lipids is relatively simple, more predictable, and can be very rich in specific fatty acids, including LC-PUFAs. Among the protists, *Crypthecodinium cohnii*, a marine heterotrophic dinoflagellate, is successfully used for the industrial production of DHA because it can accumulate DHA at high concentrations within the cells [6]. In contrast to photosynthetic microalgae, heterotrophs, such as *C. cohnii*, do not require light; hence, a high biomass density can be reached in conventional bioreactors.

The established carbon substrates for the growth of *C. cohnii* are glucose, ethanol, and acetate. Ethanol and acetate are found to be superior to glucose for the production of DHA, likely because of their short conversion pathway to acetyl-CoA, the key precursor of fatty acid synthesis [7]. No or marginal growth on sucrose, glycerol, fructose, maltose, rhamnose, arabinose, lactose, and galacturonic acid has been reported previously [6,8,9]. However, several recent papers [10,11,12] demonstrated *C. cohnii* growth and abundant DHA synthesis in glycerol. Glycerol is an interesting renewable substrate since it is a by-product of biodiesel production and other industries, and is generated globally in large quantities. The contradictory information in the literature about the consumption of glycerol by *C. cohnii*, and DHA production from glycerol, calls for a closer look at this substrate. Notably, glycerol consumption requires just two additional reactions (glycerol kinase and glycerol-3-phosphate dehydrogenase) until it enters the metabolic “highway” of glycolysis.

The systems biology approach is used to gain a mechanistic understanding of the functioning of metabolic pathways and the theoretical limitations of different biotechnologically used but insufficiently explored organisms by combining laboratory experiments and mathematical modeling [13,14,15,16]. The implementation of the systems biology approach in education and production can lead to improvements in industrial biotechnology facilitated by interdisciplinary synergy [15,17]. The applications of different modeling approaches shed light on different aspects of the process of interest [18], enabling the implementation of different types of case-specific constraints [19].

In the present work, the authors focused on the experimental work and mathematical modeling of *C. cohnii*-central metabolic fluxes with three substrates: (i) glucose, as the most widely used carbon substrate for laboratory cultivation of this dinoflagellate [6]; (ii) ethanol, reported to be the best substrate for accumulation of DHA [20]; and (iii) glycerol, as an important renewable substrate, yet with somewhat contradictory evidence on its consumption and DHA production in *C. cohnii* [9,11,12]. The enzymatic capacity of metabolic pathways towards Acetyl-CoA (DHA precursor) is analyzed by a kinetic model. The availability of metabolic resources at the central metabolism scale is assessed by a stoichiometric model. 

## 2. Results

### 2.1. Comparison of Growth, Substrate Consumption, and Accumulation of PUFAs with Glucose, Ethanol and Glycerol

**Figure 1:**
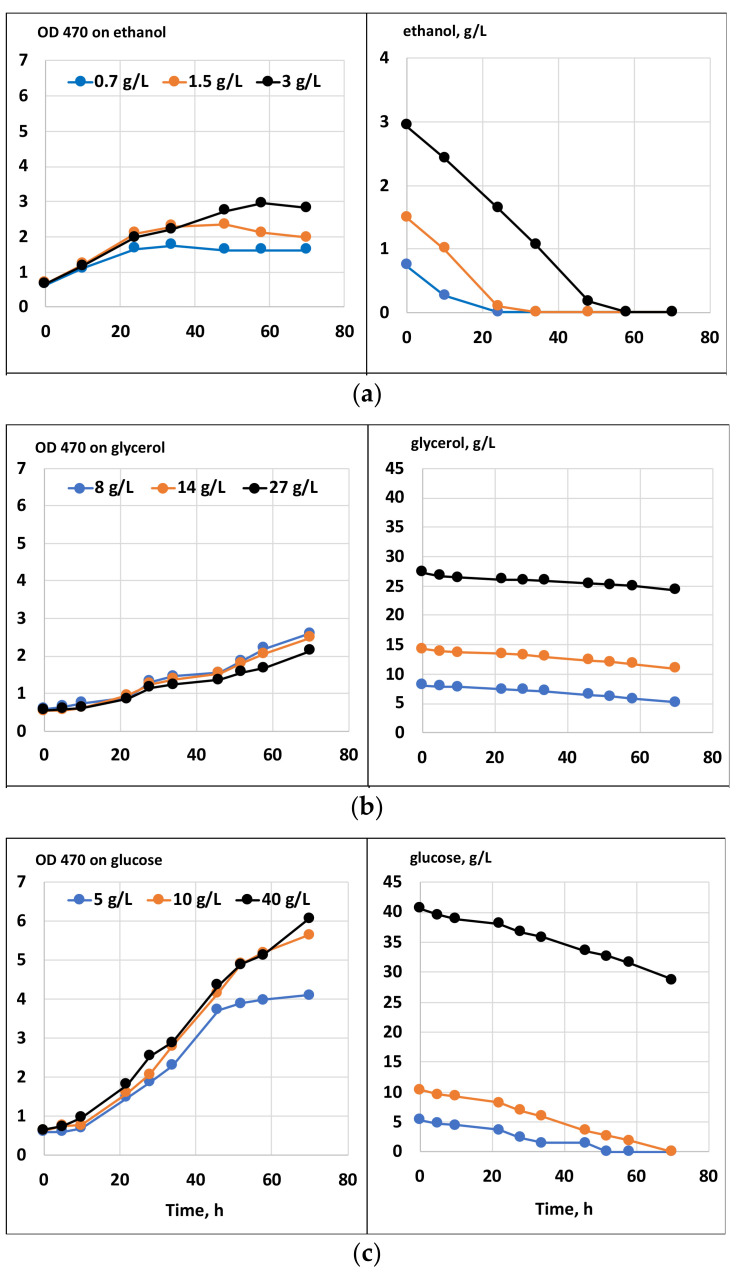
Growth and substrate consumption of *C. cohnii* on media with ethanol (**a**), glycerol (**b**), or glucose (**c**).

**Figure 2:**
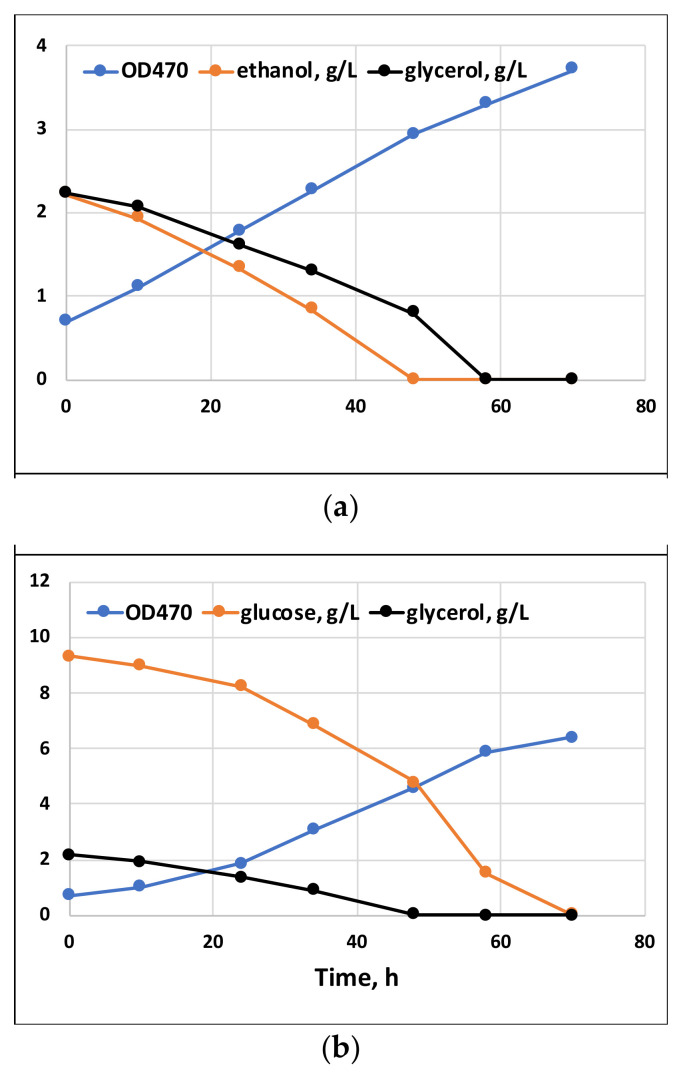
Mixotrophic growth of *C. cohnii* on glycerol with ethanol (**a**) or with glucose (**b**).

Batch cultivation results with a single carbon substrate, or their combinations with glycerol, are shown in Figure 1 and Figure 2, respectively. The growth on glycerol was compared to the growth on glucose and ethanol within a range of substrate concentrations. As seen in Figure 1, at all concentrations, the tested growth and substrate consumption with glycerol were roughly comparable to those with ethanol but proceeded significantly slower than with glucose. In contrast, Taborda et al. [12] and Safdar et al. [10] reported the growth and uptake rates for glycerol as comparable or even surpassing those with glucose. Apparently, growth parameters might vary depending on the strain, inoculum size and other cultivation parameters. Glycerol can be applied in a wide range of concentrations without any significant variations in its uptake kinetics or growth inhibition. Ethanol, in contrast, is demonstrated to inhibit growth at concentrations above 5 g/L [20]. Clearly, our data confirm that glycerol could serve as the sole carbon substrate for *C. cohnii* cultivation. At the same time, it could potentially be used as a co-substrate for mixotrophic cultivations. Under mixotrophic growth conditions (Figure 2), the uptake of glycerol and glucose occurred simultaneously, although at the initial stage of cultivation, glycerol slightly slowed down glucose consumption (compared with the growth on glucose as the sole carbon source, shown in Figure 1). Additionally, ethanol could be taken up simultaneously with glycerol (Figure 2).

**Figure 3:**
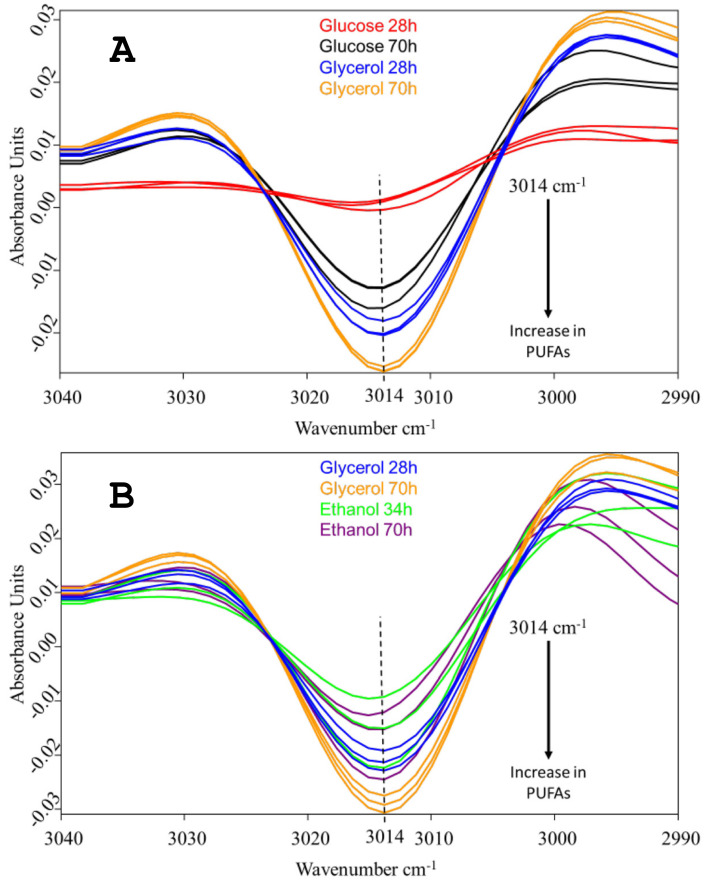
Vector-normalized, second-derivative FTIR spectra of *C. cohnii* biomass, showing relative amounts of accumulated PUFAs when grown with glycerol vs. glucose (**a**) or with glycerol vs. ethanol (**b**). Spectra obtained from cultivations with three concentrations of each carbon source are presented: with 5 g/L, 10 g/L and 40 g/L of glucose; 8 g/L, 14 g/L and 27 g/L of glycerol; and 0.7 g/L, 1.5 g/L and 3 g/L of ethanol.

The early-stage accumulation of PUFAs in the *C. cohnii* biomass, cultivated on each of the three carbon sources, was monitored by FTIR spectroscopy, following the approach used in Didrihsone et al. [21]. FTIR was chosen as a rapid analytical method, requiring a small sample size and no complex pretreatment steps. The validity of infrared spectroscopy to estimate the content of saturated, monounsaturated and polyunsaturated fatty acids has been reported previously [22,23,24]. Yoshida and Yoshida [25] evaluated the FTIR spectra of synthetic and dietary triglyceride oils with various PUFAs, including DHA. The second-derivative spectra for the alkene (-HC=CH-) C-H stretching vibrational mode of several synthetic triglycerides and dietary PUFA oils showed that the peak position corresponded to the peak position in raw spectra, and the position was changed from 3005 to 3013 cm^−1^ when the extent of unsaturation was increased from mono-ene to hexa-ene. Particularly in spectra of DHA oils, the alkene peak position was at 3013.4 cm^−1^. Here, the second-derivative spectra revealed a small peak at 3014 cm^−1^ as a simple, separate spectral feature, and accordingly, could be ascribed to the =CH- stretching of cis-alkene in PUFAs of the *C. cohnii* cells (Figure 3). The vast evidence accumulated so far on the fatty acid composition of *C. cohnii* cells indicates that DHA is the dominant PUFA in this species [26,27,28,29,30]. Apart from DHA (C22:6), there is a small amount of C22:5, while the rest of its fatty acid fraction is composed of C18:1, and of C12-C18 saturated fatty acids. Notably, DHA is the only representative of hexa-enes at measurable quantities; therefore, the spectral feature at 3014 cm^−1^ can be specifically related to *C. cohnii* DHA.

The strongest absorbance at 3014 cm^−1^ was found in the glycerol-grown cells. Notably, the accumulation of PUFAs with glycerol was already well-pronounced after 28 h of cultivation. At this early time point, hardly any absorbance was seen in the glucose-grown cells, despite the fact that glucose enabled faster growth. The absorbance of the ethanol-grown cells was more similar to that of the glycerol culture; nevertheless, after 70 h of cultivation, the glycerol-grown cells had accumulated significantly more PUFAs (Figure 3). Previously, we performed a chromatographic analysis of the fatty acid composition of *C. cohnii* CCMP 316 biomass grown in fed-batch mode with ethanol [30]. Following the same methodology, we also analyzed the DHA content of the same strain, grown in batch mode on 40 g L^−1^ glucose under conditions similar to those of the present study (unpublished data). The DHA content in these cultivations was in the range of 3.0–3.5% of the biomass dry weight. Here, this value would correspond to the black lines at the top panel of Figure 3, providing a rough absolute scale for the change of DHA content, seen in the spectra.

### 2.2. Pathway-Scale Kinetic Model of Substrate Uptake 

#### 2.2.1. Structure of the Model

**Figure 4:**
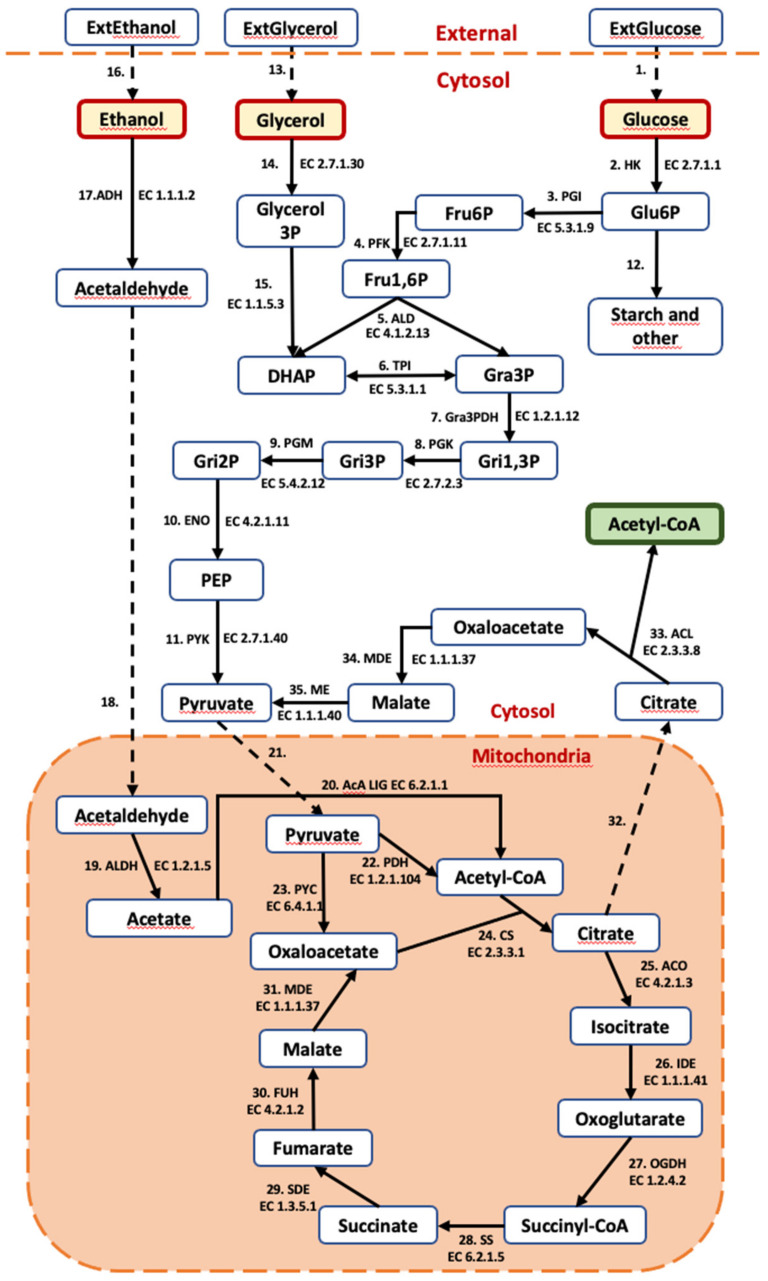
Metabolic network scope of the kinetic model. Dashed lines show transport reactions. Abbreviated metabolites—ExtGlucose: external glucose; ExtGlycerol: external glycerol; ExtEthanol: external ethanol; Glu6P: glucose 6-phosphate; Fru6p: fructose 6-phosphate; Fru1,6P: fructose 1,6-bisphosphate; DHAP: dihydroxyacetone phosphate; Gra3P: glyceraldehyde-3-phopshate; Gri1,3P: glycerate-1,3-biphosphate; Gri3P: glycerate-2-phosphate; Gri2P: Glycerate-2-phosphate; PEP: phosphoenolpyruvate; Acetyl-CoA: acetyl coenzyme-A. (Enzymes: HK: hexokinase; PGI: Phosphoglucose isomerase; PFK: Phosphofuctokinase; ALD: Fructosebiphosphate aldolase; TPI: Triosephosphate isomerase; Gra3PDH: Glyceraldehyde phosphate dehydrogenase; PGK: 3-phosphoglycerate kinase; PGM: Phosphoglycerolmutase; ENO: Phosphopyruvate hydratase; PYK: Pyruvate kinase; PDH: pyruvate dehydrogenase; PYC: pyruvate carboxylase; CS: citrate synthase; ACO: aconitate hydratase; IDE: isocitrate dehydrogenase; OGDH: 2-oxoglutarate dehydrogenase; SS: succinyl-CoA synthetase; SDE: succinate dehydrogenase; FUH: fumarate hydratase; MDE: malate dehydrogenase; ACL: ATP-dependent citrate lyase; ME: malic enzyme; ADH: alcohol dehydrogenase; ALDH: acetaldehyde dehydrogenase; AcA LIG: acetate CoA ligase).

A kinetic ordinary differential equation (ODE)-based model of *C. cohnii*, including metabolic reactions that connect glucose, ethanol and glycerol uptake and the Krebs cycle with the production of Acetyl-CoA, the precursor of DHA, was developed. The model is organized into three compartments (extracellular, cytosol and mitochondria). The model contains 35 reactions and 36 metabolites (Figure 4). 

The model structure was developed based on research by Zhang’s group on transcriptomics [31] and the ^13^C metabolic flux analysis [32] of DHA production in the case of glucose consumption. This kinetic model structure is similar to the structure proposed in Cui et al. [32]; however, the pentose phosphate pathway and glutamate dehydrogenase reactions were removed to make the kinetic model simpler and because the fluxes through these reactions were relatively small. The model does not include energy and redox cofactor moieties. The kinetic equations and some parameters of the reactions were obtained from the following databases: *Brenda* [33], *SABIO-RK* [34] and *UniProt* [35]. The tricarboxylic acid cycle reaction parameters were adapted from [36]. The equilibrium constant of reactions was assessed using *Equilibrator* [37] and the NIST database (https://randr.nist.gov/enzyme/, accessed on 3 January 2022). The unit used for the reaction fluxes in the model is mmol·L^−1^·min^−1^.

#### 2.2.2. Parameter Estimation Results

Three experimental parameter sets have been developed for the kinetic model to account for different substrate uptakes: glucose, glycerol and ethanol. The most detailed published dataset available corresponds to the consumption of glucose based on ^13^C metabolic flux analysis [32] with a glucose consumption rate of 3.58 mmoL·min^−1^·L^−1^ and reaction rates, including the Krebs cycle and Acetyl-CoA production. For modeling purposes, a single, concentration-independent substrate uptake rate for glycerol and ethanol was derived from the cultivation experiments described in Section 2.1. 

During the parameter estimation, it became clear that a single set of model parameters could not describe all three examined substrates. The same parameter set of kinetic models could be used for glucose and glycerol experiments. This could be expected because of the common pathway of glucose and glycerol from Gra3P to pyruvate, which then enters the mitochondria, serving as the precursor for both mitochondrial oxaloacetate (reaction PYC) and mitochondrial Acetyl-CoA (reaction PDH). It turned out that, in the case of ethanol that enters the Krebs cycle via Acetyl-CoA, the PDH reaction rate had to be close to zero to facilitate all of the mitochondrial pyruvate flux towards mitochondrial oxaloacetate.

As a result, we developed two structurally identical kinetic models that were able to simulate the experimentally observed data. Both models were deposited in the BioModels [38] database in SBML (level 2 version 4) and COPASI formats: (1) glucose and glycerol consumption model (Biomodels ID: MODEL2112280001) with a Vmax of PDH being 907 mmoL·min^−1^·L^−1^ (Supplementary Files S1 and S2) ethanol consumption model (Biomodels ID: MODEL2112290001) with a low Vmax of PDH 1e-6 mmol·min^−1^·L^−1^ (Supplementary File S2). The parameters of the models are summarized in Supplementary File S3.

#### 2.2.3. Simulation Results

The simulations of the glucose/glycerol model confirm the experimentally determined production flux of cellular Acetyl-CoA at 3.87 mmoL·min^−1^·L^−1^ when consuming glucose at 3.58 mmoL·min^−1^·L^−1^ (Table 1). The same model predicts the cellular Acetyl-CoA production flux at 1.44 mmoL·min^−1^·L^−1^ when consuming glycerol at 2.42 mmoL·min^−1^·L^−1^. The ethanol model predicts a cellular Acetyl-CoA production flux of 4.76 mmol·min^−1^·L^−1^ when consuming ethanol at 7.76 mmoL·min^−1^·L^−1^. This means that the percentage of substrate that undergoes carbon transformation into two carbon atoms of Acetyl-CoA is 36, 40 and 61% for glucose, glycerol and ethanol, respectively. The most efficient substrate in terms of carbon uptake (C1 moles) at the experimentally observed uptake rate is glucose (21.46 mmoL·min^−1^·L^−1^) followed by ethanol (15.52 mmoL·min^−1^·L^−1^) and glycerol (7.27 mmoL·min^−1^·L^−1^).

### 2.3. Medium-Scale Stoichiometric Model of DHA Production

#### 2.3.1. Validation of the Model

A medium-scale stoichiometric, central, carbon metabolism model of *C. cohnii* has been developed. The model is organized in three compartments (extracellular, cytosol and mitochondria) and has 398 reactions and 468 metabolites. Out of these 398 reactions, 35 are transport reactions (metabolite uptake, shuttle transport, metabolite output). The model simulates the uptake of the substrates, as well as H_2_O, O_2_, H^+^ and ammonia, which is available to the *C. cohnii* for uptake in a bioreactor. The model is available in COBRA format and MS Excel format (Supplementary File S4) and is available in the BioModels database in SBML format (Bomodels ID: MODEL2112300001).

The biomass equation was created by using biomass composition data from Cui et al. [32], determining the amount of each metabolite needed to form 1 gram of biomass [39]. To determine the ratio between the nucleotides that make up the RNA and DNA, *C. cohnii* transcriptome [31] and *Symbiodinium minutum* genome [40] data were used. The unit used for the reaction fluxes in the model is mmol·gDW^−1^·h^−1^.

The stoichiometric model was validated using published experimental results, as well as experiments performed during this study (Table 2), reaching the specific growth rate when consuming the substrate at the experimentally observed uptake rate. 

**Figure 5:**
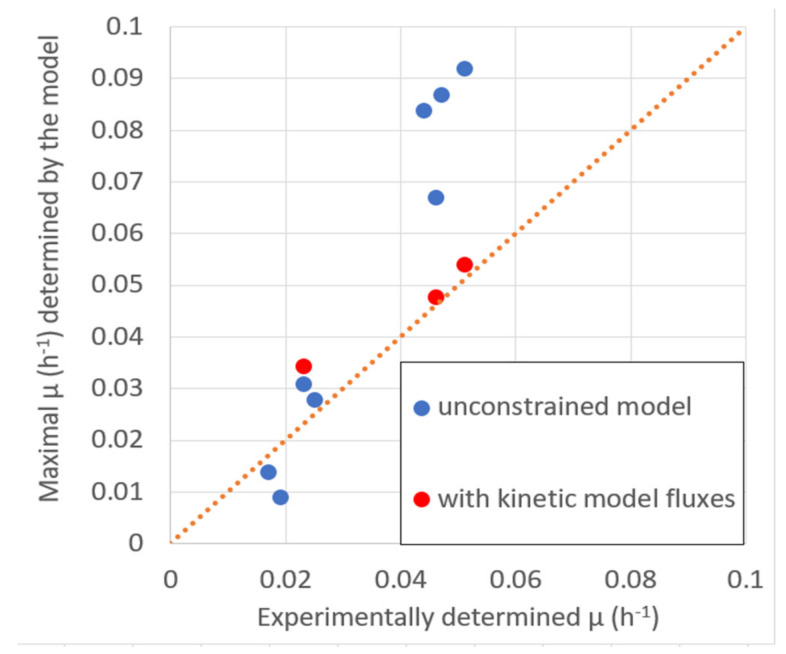
The stoichiometric model predicted the maximal specific growth rate μ_max_ comparison with experimentally determined μ at experimentally determined values of substrate consumption (Table 2).

The maximal biomass productivity with the given substrate uptake, according to validation data (Table 2), was determined by maximizing biomass production in the stoichiometric model to demonstrate that, in most cases, the μ_max_ of the model is close or higher than the experimentally observed μ (Figure 5), indicating that model predictions are close to the experimentally determined values or above them. Higher model predictions suggest that the growth in the experiment did not reach the maximal rate for unspecified reasons.

#### 2.3.2. Validation of Steady-State Fluxes of the Kinetic Model

The structure of the stoichiometric model includes all reactions of the pathway-scale kinetic model. This enables the feasibility testing of steady-state, pathway-scale kinetic model fluxes within the framework of the medium-scale stoichiometric model, namely, the biomass production at the experimentally determined substrate consumption and intracellular reaction rates of Acetyl-CoA production. This model has been validated by three steady-state flux datasets (Supplementary File S5) of simulations mentioned in Table 1.

The stoichiometric model could simulate the kinetic model steady-state fluxes of glucose consumption, largely due to the fact that the fluxes were based on ^13^C flux experimental data that covered all relevant branches (Supplementary File S5, Sheet “Glucose”). 

In the case of glycerol, the kinetic model did not take into account the flux to the pentose phosphate pathway. Therefore, larger flux values for the reactions PGI, PFK and FBA were allowed in the stoichiometric model, and the small kinetic model values in glucose and ethanol uptake were set to zero (Supplementary File S5, Sheet “Glycerol”). 

The kinetic model steady-state flux set for ethanol consumption also had to be corrected to enable the operation of the pentose phosphate pathway in a similar way, as in the case of glycerol (Supplementary File S5, Sheet “Ethanol”). The transport rates of other substrates were set to zero.

Steady states were reached with the accepted variability of some reactions up to 4% for glucose, 10% for glycerol and 3% for ethanol. This variability was introduced to compensate for potential measurement errors and to meet the full balance pre-condition of constraint-based stoichiometric modeling.

### 2.4. Model-Based Determination of DHA Production Potential

The effectivity of carbon conversion into biomass can be analyzed in several ways. We looked at the biomass production rate and the efficiency of substrate carbon transformation into biomass (Table 3). The experimentally observed biomass production rate μ is the highest in the case of glucose and the lowest in the case of glycerol. However, glycerol shows the highest efficiency of substrate transformation into biomass (57.4 mmoLC1·gDW^−1^), while glucose is the least efficient (76.5 mmoL mmoLC1·gDW^−1^). The optimization of the stoichiometric model, without taking into account the fluxes simulated by the kinetic model, reveals that any substrate of interest can be transformed into biomass with a ratio of about 42 mmoLC1·gDW^−1^. This means that the experimentally observed transformation rate of glycerol is the closest to the theoretical value using 35% more carbon than predicted by the model in an optimal case. In the case of glucose and ethanol, that is 80% and 45%, respectively.

**Figure 6:**
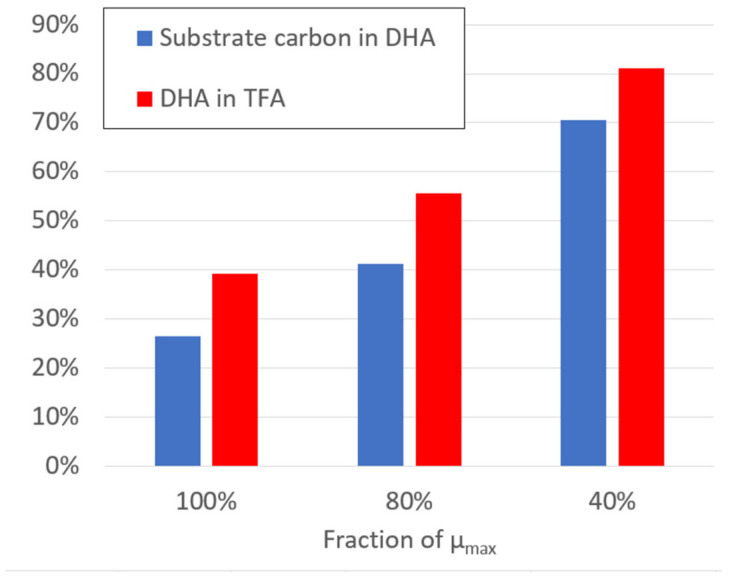
Estimation of DHA production potential by the constraint-based stoichiometric model at different biomass production intensities: 100% (=μ_max_), 80% and 40%.

The DHA production potential was determined by the stoichiometric model without taking into account the kinetic model fluxes for different biomass production intensities (Figure 6). The calculations were carried out by the stoichiometric model at experimentally observed substrate uptake rates of glucose, glycerol and ethanol. The maximal specific growth rate (μ_max_) was determined by maximizing biomass function, assuming that all substrates will be targeted at biomass production with DHA as a part of the biomass. Knowing that the DHA fraction in the experimentally produced biomass was variable (Figure 3), we introduced a DHA production reaction to simulate DHA overproduction, which increases in cases when 80% or 40% of the maximal biomass produced. Taking into account equal substrate transformation ratios into biomass, calculated numbers are equal for all substrates.

The stoichiometric model simulations indicate that DHA production potential increases when biomass production decreases. In the case of the maximal biomass production, the percentage of substrate carbon that forms DHA grows from 27% at the maximal biomass production rate up to 70% in the case of 40% of maximal biomass production rate. The percentage of DHA in total fatty acids (TFA) increases from 39% to 81%, respectively. These calculations are based on the assumption that all metabolic resources that do not form biomass are directed by the available metabolic reactions towards the production of DHA.

## 3. Discussion

### 3.1. Combining Kinetic and Stoichiometric Models

In the present study, we created kinetic and stoichiometric models of *C. cohnii*-central metabolism that are validated by ^13^C fluxomic data [32]. The knowledge of the involvement of central metabolism reactions in the transformation of substrates to DHA is extended by the interaction of pathway-scale kinetic and constraint-based stoichiometric models to a larger scale, thus enabling a narrower scope of feasible metabolic scenarios. 

Kinetic models usually cover a pathway-scale number of reactions [41]. Kinetic models contain a mathematical description of the kinetics of reaction mechanisms such as the Michaelis–Menten reaction, mass action and others. This type of model provides an opportunity to quantitatively simulate the values of metabolite concentrations and reaction fluxes. In kinetic modeling, it is optimistically assumed that the necessary energy, redox cofactor and some other metabolites are supplied by the remaining metabolism in some way [19].

In contrast to kinetic models, stoichiometric models require fewer details for individual reactions and, as a consequence, can be applied at the genome-scale [42,43]. The stoichiometric approach can be used for the analysis of feasible steady states, provided that there is information about the reaction stoichiometry. The advantage of stoichiometric models is their ability to find out whether all of the involved metabolites have precursors supplied for their production [19]. In the present work, we combined both modeling approaches [44].

The combination of both models enabled the feasibility of internal fluxes, which were calculated by the kinetic models of ethanol and glycerol, to be tested; they have never been measured experimentally. The test resulted in a rejection of some steady-state fluxes that were suggested by the kinetic model in the case of glycerol and ethanol, showing the usefulness of the iterative application of both model types. Steady-state fluxes that were kinetically feasible in the ODE-based model became unfeasible in the constraint-based stoichiometric model, where all biomass compounds had to be produced in a specific proportion. Thus, the stoichiometric model demonstrated that some fluxes simulated by the kinetic model disabled the production of all necessary metabolites in parallel with the production of biomass at the experimentally observed specific growth rate, suggesting the necessity for additional experiments to determine feasible flux distributions. 

### 3.2. Analysis of Substrate-Specific Functioning of Central Metabolism by Experimental and Modeling Analysis

Our aim here was to employ the model simulation of central metabolism for a better understanding of the conversion of several substrates into the target product: DHA. In particular, we were interested in glycerol as a potential renewable for the synthesis of PUFAs, still poorly studied as a substrate for growth and DHA production in *C. cohnii*. We used both model types to establish (1) if the enzymatic capacity ensured the sufficient supply kinetics of Acetyl-CoA, the key central metabolite needed for DHA production, and (2) if there was the required number of metabolic precursors available for the building blocks of DHA. So far, this kind of approach has not been applied in the analysis of *C. cohnii* or any other dinoflagellates.

Both kinetic and stoichiometric models were used to simulate the observed kinetics for the uptake of glucose, ethanol and glycerol, with a particular focus on the early stages of culture growth. Kinetic and stoichiometric models were able to simulate the experimental observations (Table 2). 

At the level of the pathway-scale kinetic model, it was found that the functioning of the model with ethanol as the substrate was only possible if the PDH reaction did not operate (V_max_ of PDH is close to zero). The necessity to block the reaction in the case of ethanol is determined by the fact that, in contrast to the glucose and glycerol pathways, the ethanol catabolic pathway produces mitochondrial Acetyl-CoA, and all pyruvate pools should be redirected for the regeneration of mitochondrial oxaloacetate to provide the acceptor for the CS reaction. There are several possible mechanisms for the heavy reduction in PDH flux: (1) the allosteric inhibition of PDH by Acetyl-CoA [45]; (2) the covalent modification by phosphorylation with ATP [46], and (3) the regulation of PDH expression at the transcriptional level. PDH allosteric inhibition by Acetyl-CoA seems most likely since it is supported by the kinetic model, showing higher concentrations of mitochondrial Acetyl-CoA in the case of ethanol consumption (2.1 × 10^−3^ mmoL·L^−1^) than when consuming glucose (4.5 × 10^−4^ mmoL·L^−1^) or glycerol (1.8 × 10^−3^ mmoL·L^−1^).

We found that with glycerol, the cells grew slower than with glucose yet tended to accumulate more PUFAs, than with both other substrates (Figure 3). This is supported by the experimental observations that the carbon from glycerol is more efficiently transformed into biomass (Table 3). Potentially, another reason why glycerol is advantageous for DHA accumulation might be related to the storage of DHA in the cells. Most of the DHA in *C. cohnii* is incorporated in triacylglycerols [9]. Therefore, as DHA is being produced, part of the available glycerol could be directly utilized for triacylglycerol synthesis, removing the free DHA, and thus stimulating its synthesis. When growing on glucose or ethanol, the supply of glycerol for triacylglycerol synthesis requires additional metabolic reactions and might represent a bottleneck.

Crude glycerol, derived from biodiesel production, contains inhibitory substances, and its utilization for food-grade DHA production poses problems, as previously analyzed by Sijtsma et al. [9]. However, an unexpected observation was recently reported by Taborda et al. [12]. These authors found that crude glycerol was superior to pure glycerol with respect to DHA yields and productivity and was comparable to glucose. This might have far-reaching practical applications, yet still requires a more detailed study. 

The fact that the stoichiometric model could find ways to produce DHA equally well from carbon supplied by any of the analyzed substrates indicates that some details of metabolism (inhibition due to substrate concentration or enzyme capacity limitations and other factors), which are not included in the stoichiometric model, would make the substrate conversion rate closer to the experimentally observed conversion rates. Unfortunately, a genome-scale, constraint-based stoichiometric model cannot be developed at this moment as no genome sequence of *C. cohnii* has been published.

The assumption that all free metabolic resources are targeted towards DHA production is introduced for the estimation of the production potential of DHA. Metabolic engineering [47] is needed to find out what fraction of the potential determined by the model is reachable in praxis.

The combination of ODE-based, pathway-scale kinetic modeling and constraint-based stoichiometric modeling with ^13^C data enables a more detailed insight into the flux distribution within the organism. The combined application of different types of models enables the rejection of many unfeasible hypotheses that may arise due to the limited predictivity of each separate modeling type. Both models and their combinations can be used to explore a wider range of problems in metabolism and its optimization.

## 4. Materials and Methods

**Figure 7:**
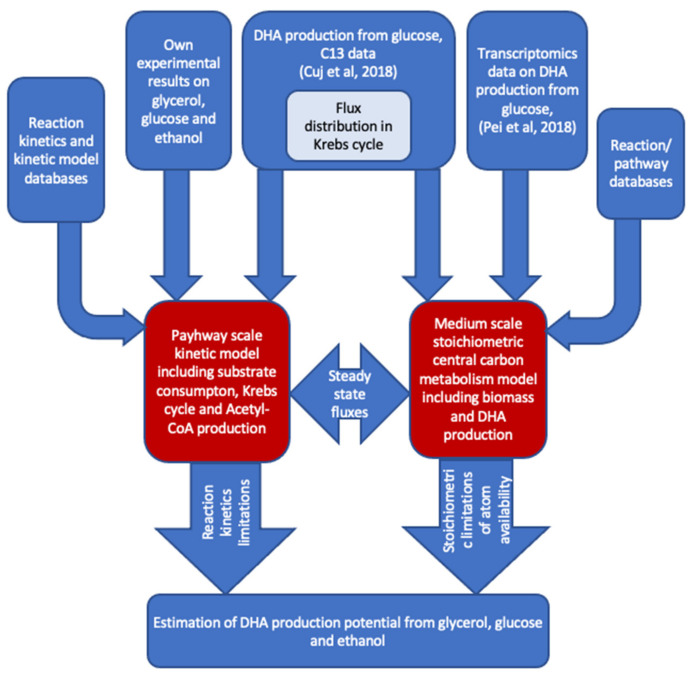
Information flow-to and -from pathway-scale kinetic model and central carbon metabolism scale constraint-based stoichiometric model.

To explore the potential of DHA production from glycerol, glucose and ethanol, the authors combined literature data, their own experimental results, the pathway-scale kinetic model and medium-scale stoichiometric model (Figure 7). The ^13^C data on the DHA production from glucose [32] were used to parametrize the Krebs cycle of kinetic and stoichiometric models. After that, the substrate consumption rates with corresponding biomass production rates were used to find out the potential amount of DHA that could be produced from the particular substrate. Pathway-scale kinetic models contributed here with detailed kinetic rate equations for substrate-specific transport and metabolic reactions assessing the sufficiency of the enzymatic capacity of reactions and transports. The medium-scale stoichiometric model takes into account the main duties of central metabolism to produce biomass with balanced reactions, leading to a full accounting of all elements of reactions to establish the availability of all molecules that apply balanced reactions. The DNA production potential is estimated by the stoichiometric model by fixing biomass production at a reasonable level and maximizing DHA production from the selected substrate. 

### 4.1. Experimental Materials and Methods

Culture maintenance and cultivations were performed on a medium with sea salts and yeast extract, as described previously [30]. In brief, *Crypthecodinium cohnii* CCMP 316 was obtained from the National Center for Marine Algae and Microbiota, USA. It was cultivated on a complex medium containing 2 g L^−1^ yeast extract, 25 g L^−1^ sea salt (Sigma-Aldrich) and various concentrations of glycerol, glucose and/or ethanol, as specified in the Results section. Cultivations were carried out aerobically at 25 °C in 0.5 L or 1 L Erlenmeyer shaken flasks with 200 mL of culture on a rotary shaker at 140–180 r.p.m. The concentrations of glucose, ethanol and glycerol in culture media were monitored by HPLC, as described previously [30,48].

FTIR spectra of algal biomass were recorded using Vertex 70 coupled with the microplate reader HTS-XT (Bruker, Germany). Spectra were recorded in the frequency range of 3800–600 cm^−1^, with a spectral resolution of 4 cm^−1^, and 64 scans were coadded. Only spectra with absorbance within the absorption limits between 0.25 and 0.80 (where the concentration of a component is proportional to the intensity of the absorption band) were used for data analysis. The FTIR spectra were vector normalized and deconvoluted (second derivative) for more precise evaluation of weak-intensity spectral bands and to resolve the overlapping components, if any [49]. Data were processed using OPUS 7.5 software (Bruker Optics GmbH, Ettlingen, Germany). The baseline of each spectrum was corrected by the rubber band method.

### 4.2. Development of a Pathway-Scale Kinetic Model

The model was developed in *COPASI* (COmplex PAthway SImulator) simulation software [50,51] version 4.34 (Build 251). The estimation for kinetic equation parameters that were not found in literature or databases was conducted using built-in parameter estimation functionality using global stochastic optimization methods. The model-specific parameter estimation performance of global stochastic optimization methods implemented in *COPASI* was tested using *ConvAn* software [52]. During parameter estimation, multiple parallel optimization runs were applied, using *COPASI* wrapper *SpaceScanner* [53] to select the most efficient global stochastic optimization algorithms, reducing misinterpretation risks of optimization results [54]. The total concentration of used amino acids in the reactions included in the model was limited to avoid unnecessarily high enzyme concentrations that would not be evolutionarily favorable.

Model parameters were either obtained from the literature or inferred from experimental data. An additional parameter *V_m_* was added to the reactions used from [36] to change the *V*_max_ of these reactions without changing the *V*_f_ to *V*_r_ ratios. Kinetic equations of all enzymatic reactions had overexpression coefficients k that could be used for optimizing enzyme concentrations to increase Acetyl-CoA or other molecule production. Currently, all coefficients *k* = 1 so that the model corresponds to wild-type concentrations of enzymes.

Species concentration constraints were applied in the parameter estimation task in *COPASI*. From Park et al. [55], it was implemented as a constraint that the metabolite concentrations in this model should not exceed 12 mmol/L, except for cellular ethanol, which was allowed to reach 32 mmoL/L.

The metabolic flux unit in the kinetic model is mmoL·min^−1^·L^−1^ since it is frequently used in kinetic models, while in the stoichiometric model, the metabolic flux unit is mmoL·gDW^−1^·h^−1^. To transition from dry-weight-related measurements to absolute weight, it was assumed that dry weight made up 33% of the absolute weight and the cell density was 1 g·mL^−1^. Mitochondrial volume was made to be 1% of the cytosol volume [56].

To determine the parameters that were dependent on enzyme concentrations, three sets of experimental data were used. The first set included reaction fluxes adapted from Cui et al. ^13^C metabolic flux analysis for growth on glucose. The second and third sets included experimentally measured glycerol and ethanol consumption rates (Section 2.1) determined during this study. The experimental data of glucose consumption were not used in parameter estimation due to the high similarity with ^13^C experimental data. The *Parameter estimation* task was used in *COPASI*; the data sets were added as different experiments. 

### 4.3. Development of the Constraint-Based Medium-Scale Stoichiometric Model

A constraint-based, medium-scale stoichiometric model [57] of central carbon metabolism, biomass production and pathways to DHA was developed, extending the scope of the kinetic model. Reactions were included in the model based on the results of transcriptomics [31] and on the reactions from the genome annotation of the *Symbiodinium minutum* genome, which is a phylogenetically close relative of *C. cohnii*. The specific growth rate was calculated by an exponential approximation of the growth curve. It is assumed that DHA production is constant during the growth period.

The model was built and optimized using COBRA Toolbox v3.0 [58] and RAVEN 2.0 [59] functionality. The model was visualized by Paint4Net [60], Escher [61] and IMFLer [62] software.

The model was validated using the experimental data generated during this study and those found in the literature.

## 5. Conclusions

Kinetic and stoichiometric modeling-based analysis demonstrates the attractiveness of glycerol as a substrate for DHA (main fraction of PUFA [30]) production by *C. cohnii*, along with established substrates, such as ethanol and glucose. This is proven experimentally and analyzed mathematically by mechanistic models of *C. cohnii* metabolism. The promising results on the applicability of crude glycerol [12] increase the attractivity of glycerol even further. 

The iterative application of the pathway-scale kinetic model and constraint-based stoichiometric model combines the accuracy of the kinetic model of the main product-forming pathways with the large-scale stoichiometric model’s ability to determine if the pathways addressed by the kinetic model could be supplied with all of the necessary molecular components. Simultaneously, biomass could be produced by the metabolic network of the organism of interest. This approach is important for improving the understanding of metabolic network functionality and increasing the predictability and efficiency of metabolic engineering efforts.

Our experiments, in combination with modeling, supported the potential of glycerol as another renewable substrate of *C. cohnii* for the production of DHA. Despite a lower consumption rate and lower specific growth rate, the PUFA content and efficiency of carbon transformation into biomass are better with glycerol than with glucose. Therefore, the sustainability parameters [63] of DHA production from glycerol are expected to be better than in the case of glucose and ethanol.

## Figures and Tables

**Table 1:** Some simulated flux rates for different substrates.

Experimental Data	Substrate Concentration mmoL·L^−1^	Substrate Uptake mmoL·min^−1^·L^−1^	Single Carbon (C1) Uptake mmoL·min^−1^·L^−1^	Krebs Cycle Flux mmoL·min^−1^·L^−1^	ACL EC 2.3.3.8 Flux mmoL·min^−1^·L^−1^	Specific Growth Rate μ h^−1^
Cui et.al. 2018 [32]	Glucose, up to 50	3.58	21.46	2.43	3.87	0.051
This study	Glycerol, up to 130	2.42	7.27	0.90	1.44	0.023
This study	Ethanol, up to 32	7.76	15.52	3.00	4.76	0.046

**Table 2:** Validation data.

Reference	Consumption mmoL·gDW^−1^·h^−1^	Specific Growth Rate μ h^−1^
Cui et.al. 2018 [32]	Glucose 0.65	0.051
Cui et.al. 2018 with ETA [32]	Glucose 0.61	0.047
This study	Glucose 0.59	0.044
Taborda et al. 2021 [12]	Glucose 0.37	0.017
This study	Glycerol 0.44	0.023
Taborda et al. 2021 [12]	Glycerol 0.43	0.019
This study	Ethanol 1.41	0.046
Taborda et al. 2021 [12]	Acetate 0.60	0.025

**Table 3:** The efficiency of substrate transformation into biomass for experimentally observed and optimized data.

Experimental Data	Substrate Uptake mmoL·gDW^−1^·h^−1^	Carbon (C1) Uptake mmoL·gDW^−1^·h^−1^	Experimental		Optimized by Stoichiometric Modeling	
			μ h^−1^	Carbon C1 per gDW Biomass mmoL·gDW^−1^	μ_max_ h^−1^	Carbon C1 per gDW Biomass mmoL·gDW^−1^
Cui et.al. 2018 [32]	Glucose 0.65 (=3.58 mmol·min^−1^·L^−1^)	3.9	0.051	76.5	0.092	42.4
This study	Glycerol 0.44 (=2.42 mmol·min^−1^·L^−1^)	1.32	0.023	57.4	0.031	42.6
This study	Ethanol 1.41 (=7.76 mmol·min^−1^·L^−1^)	2.82	0.046	61.3	0.067	42.1

## Data Availability

Data is contained within the article or supplementary material.

## Supplementary Materials

The following are available online at https://www.mdpi.com/article/10.3390/md20020115/s1, File S1: Kinetic model for Acetyl-CoA production from glucose and glycerol by *C. cohnii*, File S2: Kinetic model for Acetyl-CoA production from ethanol by *C. cohnii*, File S3: Parameters of kinetic models for Acetyl-CoA production from glucose, glycerol and ethanol by *C. cohnii*, File S4: Constraint-based stoichiometric model of central metabolism of *C. cohnii*, File S5: Flux distribution of kinetic models for Acetyl-CoA production from glucose, glycerol and ethanol (columns A–C and F). The supplementary information includes also flux values adapted for stoichiometric model simulations (columns D and E). Reactions marked by yellow are reactions, which are set to zero in stoichiometric model. Reactions marked by green are set to default upper and lower bounds (1000 and −1000 respectivelly).
